# Experimental designs for a Benign Paroxysmal Positional Vertigo model

**DOI:** 10.1186/1742-4682-10-21

**Published:** 2013-03-19

**Authors:** Santiago Campos‐Barreiro, Jesús López‐Fidalgo

**Affiliations:** 1 Department of Mathematics, Institute of Mathematics applied to Science and Engineering University of Castilla‐La Mancha Avda, Camilo José Cela 3, 13071‐Ciudad Real, Spain

**Keywords:** BPPV, *c*‐optimal design, *D*‐optimal design, Efficiency, Information matrix

## Abstract

**Background:**

The pathology of the Benign Paroxysmal Positional Vertigo (BPPV) is detected by a clinician through maneuvers consisting of a series of consecutive head turns that trigger the symptoms of vertigo in patient. A statistical model based on a new maneuver has been developed in order to calculate the volume of endolymph displaced after the maneuver.

**Methods:**

A simplification of the Navier‐Stokes problem from the fluids theory has been used to construct the model. In addition, the same cubic splines that are commonly used in kinematic control of robots were used to obtain an appropriate description of the different maneuvers. Then experimental designs were computed to obtain an *optimal* estimate of the model.

**Results:**

*D*‐optimal and *c*‐optimal designs of experiments have been calculated. These experiments consist of a series of specific head turns of duration *Δt* and angle *α* that should be performed by the clinician on the patient. The experimental designs obtained indicate the duration and angle of the maneuver to be performed as well as the corresponding proportion of replicates. Thus, in the D‐optimal design for 100 experiments, the maneuver consisting of a positive 30° pitch from the upright position, followed by a positive 30° roll, both with a duration of one and a half seconds is repeated 47 times. Then the maneuver with 60° /6° pitch/roll during half a second is repeated 16 times and the maneuver 90° /90° pitch/roll during half a second is repeated 37 times. Other designs with significant differences are computed and compared.

**Conclusions:**

A biomechanical model was derived to provide a quantitative basis for the detection of BPPV. The robustness study for the D‐optimal design, with respect to the choice of the nominal values of the parameters, shows high efficiencies for small variations and provides a guide to the researcher. Furthermore, c‐optimal designs give valuable assistance to check how efficient the D‐optimal design is for the estimation of each of the parameters. The experimental designs provided in this paper allow the physician to validate the model. The authors of the paper have held consultations with an ENT consultant in order to align the outline more closely to practical scenarios.

## Background

### Introduction

First described by Bárány [1], Benign Paroxysmal Positional Vertigo (BPPV) is the most common vestibular disorder leading to vertigo. These vestibular symptoms are precipitated when the orientation of the head or body is changed relative to gravity, provoking brief periods (2‐3 minutes) of vertigo, imbalance, and nausea. These changes can occur during daily activities such as lying down in bed or reaching up to retrieve an object from a high shelf. Benign Paroxysmal Positional Vertigo is commonly called *top‐shelf vertigo*[2].

**Figure 1:**
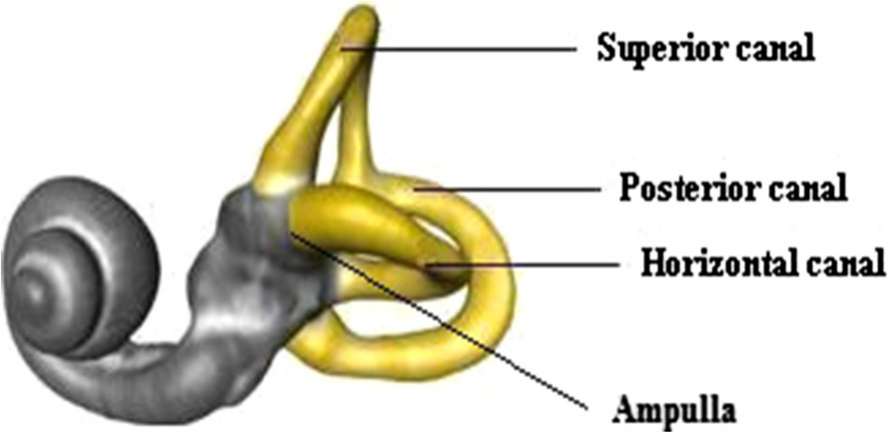
**Semicircular canals and ampulla.** Layout of the semicircular canals and ampula, both located in the inner ear.

Contrary to what is widely believed, this type of vertigo is caused by a disorder in an organ of the inner ear, called the *semicircular canal*, which regulates balance. Figure 1 illustrates the three canals and how they are arranged in a similar way to the three cartesian axes. Each canal is filled with a fluid called *endolymph* and contains motion sensors within the fluids. At the base of each canal, the bony region of the canal has a dilated sac at one end called the *ampulla*. Within the ampulla is a mound of hair cells and supporting cells called *crista ampullaris*. These hair cells are composed of many cilia and are embedded in a gelatinous structure called *cupula*. As the head rotates, the duct moves, but the endolymph lags behind. This deflects the cupula and bends the cilia within. The bending of these cilia alters an electric signal that is transmitted to the brain, which sends this information to the eyes, provoking the corresponding vestibular movement which helps us keep our balance.

When does BPPV occur? Semicircular canals are identified as the origin of BPPV. There, calcium carbonate particles (*CaCO*_3_) called *otoliths*, which are normally affixed to the canal walls, are detached by the aforementioned head or body changes. This extra mass floating in the endolymph causes an abnormal movement of the cupula, since these particles displace more volume of endolymph than usual. The brain misinterprets this displacement and sends erroneous information to the eyes, provoking a characteristic ocular nystagmus and the subsequent vertigo.

Dix and Hallpike [3] were pioneers in developing maneuvers which led to the detection of BPPV. These maneuvers consist of a series of consecutive head turns that trigger ocular responses in the patient, on the basis of which clinicians can determine whether a patient suffers from BPPV or not. Rabbit [4] developed a model which calculates the volume of endolymph displaced when the Dix and Hallpike maneuver is put into practice. In this model, a mathematical approximation consisting of a curve crossing the different angular positions was used. This is a theoretical model never validated with real data as far as the authors know. In this paper, the model is particularized to a specific real situation and an experimental plan is produced. In our case, a maneuver composed of two consecutive turns has been developed: a positive pitch (turning the head back) from the upright position, followed by a positive roll (turning the head right), as these are the most common two head movements that trigger the above‐mentioned nystagmus. In order to reflect a more realistic situation, a cubic interpolation between points has been carried out. Another advantage of carrying out the cubic interpolation with respect to Rabbit’s model, is to obtain an analytical expression of the curve. This will permit us to design statistical experiments aimed at deriving an optimal estimation of the unknown parameters of the model.

### Optimal experimental design

One of the main aims of Statistics consists of modeling the behaviour of any stochastic process or particular system. In order to carry this out, a mathematical model is always needed to explain the results obtained once the process has occurred, and predict accurately the future behaviour of that process or system. The mathematical models used in real situations depend on unknown parameters. In order to describe the way in which the results are expected to vary, we need to estimate these parameters with optimal accuracy. For that purpose, the Design of Experiments is used to help us design how to change the inputs of processes in order to observe and identify the reasons of the changes observed in the response *y*, which is usually expressed as follows,

(1) y=η(z,θ)+ϵ,

where *z* represents the set of observations to collect and *θ* the unknown parameters of the model. The error *ϵ* follows a Gaussian distribution with mean 0 and constant variance *σ*^2^, that is

$$\text{E(y)}=\eta (z,\theta )\;\text{and}\;\text{var(y)}=\sigma^{2}.$$

The problem of determining which set of observations to collect is what will define the design. It is common to say that the input variables are controlled by the researcher, while the unknown parameters are determined by nature. Optimal Design of Experiments theory allows us to find the *best* design in the sense of obtaining an optimal estimate of the parameters of the model. Next, basic concepts of this theory will be briefly presented as well as the two main criteria to obtain this *optimal* estimation. Following that, we will explain how the model for the maneuver has been constructed and finally, optimal designs for this model are calculated both for discrete and continuous design space.

An *exact experimental design* of size *N* consists of a set of *N* observations collected at points *z*_1_,…,*z*_*N*_, in a given compact space *χ*. Some of these *N* points may be repeated, meaning that several observations are taken at the same value of *z*. This number of observations is usually predetermined by experimental cost constraints. A convenient way to understand designs is to treat them as a collection of different points of *χ*, together with the proportion of the *N* observations to be allocated at each different point. This suggests the idea of extending the definition of a design to any probability measure *ξ* on *χ* (*approximate design*), that is

(2) $$\xi =\left\{\begin{array}{cccc} z_{1} & z_{2} & \cdots & z_{k}\\ p_{1} & p_{2} & \cdots & p_{k} \end{array}\right\},\;\;\text{with}\;\;\overset{k}{\underset{i=1}{\sum }}p_{i}=1.$$

The collection of weights, *p*_*i*_=*ξ*(*z*_*i*_), provide a probability measure on *χ* supported on the points *z*_1_,…,*z*_*k*_. Thus, an experiment will be replicated about *Np*_*i*_ times on value *z*_*i*_. Kiefer [5] pioneered this approach, and its many advantages are well documented in design monographs, see Silvey [6] for example. This approach has been applied to optimal treatment allocation [7], optimal estimation of kinetic parameters of the Michaelis‐Menten model [8] and the Arrhenius equation [9]. In what follows, the approximate design approach is adopted without loss of generality, restricting the attention to designs with a finite set of support points. For convenience, the design is described using a two‐row matrix, with the support points displayed in the first row and their corresponding proportions of observations in the second row (2).

Let *θ*^*T*^ be the unknown parameter vector, and let

(3) f(z)=∂η(z,θ)/∂θ,

be evaluated at the nominal value of the parameter *θ*^(0)^. This nominal value usually represents the best guess for the parameters *θ* at the beginning of the experiment and it is necessary to proceed as one would with a non‐linear model [10]. Assuming model errors follow a normal distribution and apart from an unimportant multiplicative constant, the Information Matrix of a design *ξ* is given bydisplay

$$M(\xi ,\theta )=\underset{i}{\sum }\;\xi (z_{i})\;f(z_{i})f^{T}(z_{i}),$$

where *p*_*i*_=*ξ*(*z*_*i*_) is the proportion of observations to be taken at point *z*_*i*_ (see e.g.[10]). The covariance matrix of the least squares and maximum likelihood estimator $\hat{\theta }$ is asymptotically proportional to the inverse of this matrix [6]. The use of this matrix is very important when it comes to designing the experiment in an optimal way.

The objective to be achieved is to find a design which gives the best estimation of the parameters (or linear functions of them), usually by using the least squares method or maximum‐likelihood estimation method. Through what it is defined as *criteriaΦ*, we will be able to measure the accuracy of the design and to compare different designs of the same model.

The design criteria used in this work for estimating the model parameters are *D*‐optimality and *c*‐optimality [11]. The *D*‐optimality criterion minimizes the volume of the confidence ellipsoid of the parameters and is given by *Φ*_*D*_[*M*(*ξ*,*θ*)]=det*M*(*ξ*,*θ*)^−1/*m*^, where *m* is the number of parameters in the model. The D‐optimal design will be that which minimizes the function *Φ*_*D*_[*M*(*ξ*,*θ*)]. The *c*‐optimality criteria is used to estimate a linear combination of the parameters, say *c*^*T*^*θ*; it is the variance of this estimate which is *Φ*_*c*_[*M*(*ξ*,*θ*)]=*c*^*T*^*M*^−1^(*ξ*,*θ*) *c*. It is known that these criteria are all convex and nonincreasing functions of the designs and so, designs with small criterion values are desirable [6]. A design that minimizes one of these functions *Φ* over all the designs on *χ* is called a *Φ*‐optimal design, or more specifically, a *D*‐ or a *c*‐optimal design, respectively.

An advantage of working with approximate designs is that the optimality of a particular design can be checked mathematically. Since the criteria are convex, standard convex analysis arguments using directional derivatives when *Φ* is differentiable [6], show that a design *ξ*^∗^ is *Φ*‐optimal, if and only if, it satisfies (Equivalence Theorem)

(4) $$f^{T}(z)\nabla \Phi (\xi^{*})f(z)-\text{tr}\left[\nabla \Phi (\xi^{*})M(\xi^{*},\theta )\right]\geq 0,\;z\in \chi ,$$

where *Φ*(*ξ*^∗^) is *Φ*[*M*(*ξ*^∗^,*θ*)] for short and ∇*Φ*(*ξ*^∗^) denotes the gradient of *Φ*(*ξ*^∗^). The equality is reached at the support points of *ξ*^∗^. When *Φ*=*Φ*_*D*_, that is, the *D*‐optimality criterion, ∇*Φ*(*ξ*^∗^)=*M*^−1^(*ξ*^∗^,*θ*) and *tr*[∇*Φ*(*ξ*^∗^)*M*(*ξ*^∗^,*θ*)]=*m*, where *m* is the number of parameters. Therefore, the inequality (4) becomes

$$-f^{T}(z)M^{-1}(\xi^{*},\theta )f(z)+m\geq 0,\;z\in \mathrm{\chi .}$$

The function *f*^*T*^(*z*)*M*^−1^(*ξ*^∗^,*θ*)*f*(*z*) is known as the *generalized variance*. This important theorem provides methods for constructing optimal designs [11,12].

## Derivation of a model for the maneuver

The model to be derived will be used to predict the endolymph volume displacement in response to a maneuver composed by two consecutive turns: a positive pitch (turning the head back) from the upright position followed by a positive roll (turning the head right). The standard definitions are used for head rotations: *pitch* rotates about an axis out the ear (y‐axis), *roll* rotates about an axis out the eye (x‐axis) and *yaw* rotates about an axis pointing out the top of the head (z‐axis). We are only concerned with the horizontal canal since usually the particles are located inside that area [13]. Therefore, considering *Q*(*Δt*,*α*,*θ*) to be the volume of endolymph displaced after the first turn, where *Δt* and *α* are the variables which represent duration and angle of each turn, respectively, our model is formulated as follows,

(5) y=Q(Δt,α,θ)+ϵ,Δt∈[0.5,1.5],α∈[Π/6,Π/2].

The error *ϵ* follows a Gaussian distribution with mean 0 and constant variance *σ*^2^, that is

$$\text{E(y)}=Q(\mathrm{\Delta t},\alpha ,\theta )\;\text{and}\;\text{var(y)}=\sigma^{2}.$$

The forces acting on the endolymph due to the viscous interaction with a free‐floating particle inside the canal are the interaction drag force *F* between the particle and endolymph and the gravity force $\vec{g}$ acting on the particle. After considering the forces mentioned, as well as the simplifications corresponding to the fluid, the Navier‐Stokes equations must be applied with the corresponding boundary and initial conditions [14]. Thus, the equations which relate the volume flow rate of endolymph to the pressure and inertial forces at time *t* are

(6) $$\left\{\begin{array}{l} \theta_{1}\frac{\partial }{\mathrm{\partial t}}Q(t,\alpha ,\theta )+\theta_{2}Q(t,\alpha ,\theta )=F_{i}+F_{n},\\ Q(0,\alpha ,\theta )=0. \end{array}\right)$$

The unknown parameters, *θ*^*T*^ = (*θ*_1_,*θ*_2_), are related to the damping and stiffness of the canal, respectively. The right side of the first equation is represented by two forces. The inertial forcing due to the angular acceleration of the head‐fixed system relative to the inertial frame (ground‐fixed system) is

$$F_{i}=\overset{l_{n}}{\underset{0}{\int }}\rho \;(\ddot{\vec{\Omega }}(t)\wedge \vec{R}(s))\;\vec{\text{d}s},$$

where $\ddot{\vec{\Omega }}(t)\wedge \vec{R}(s)$ stands for the tangential acceleration, $\ddot{\vec{\Omega }}(t)$ being the angular acceleration of the head relative to the ground‐fixed inertial frame resolved into the head‐fixed frame and $\vec{R}(s)$ the vector running from the head‐fixed coordinate system’s origin to the centerline of the canal. The parameterization of $\vec{R}(s)$ is made with respect to the arc length *s* (also called *natural parameter*). The head‐fixed coordinate system was defined when the subject was in the upright position prior to movement of the head. The constant *ρ* stands for the density of the canal and *l*_*n*_ is the length covered by the otolith.

The second term, *F*_*n*_, is the result of the interaction drag forces due to the particle moving relative to the fluid,

$$F_{n}=\;\frac{A_{s}\;N}{A_{e}}\left[\frac{4}{3}a(\rho_{s}-\rho_{e})(\vec{g}-\ddot{\vec{\Omega }}(t)\wedge \vec{R}(s))\cdot \vec{n}+\frac{6}{a}\mu \left(\dot{\xi }-\frac{\frac{\partial }{\mathrm{\partial t}}Q(t,\alpha ,\theta )}{A_{e}-A_{s}}\right)\right].$$

In this equation, $\;\vec{g}\;$ is the gravitational acceleration, $\vec{n}$ is the unit normal tangent vector to the canal centerline and $\dot{\xi }$ is the velocity of the particle. The constants *A*_*s*_, *N*, *A*_*e*_, *a*, *ρ*_*s*_, *ρ*_*e*_ and *μ*_*e*_, stand for frontal area of the particle, number of particles which are floating inside the canal, cross‐sectional area of the canal, radius of the particle, density of the particle, density of the endolymph and endolymph viscosity, respectively.

The manner in which the maneuver has been carried out was established by the clinician. The angle of turn in each time *t* is represented by *Ω*(*t*). The movements of the head determine the magnitude and direction of the vectors $\vec{\Omega }(t)$ and $\vec{g}$. It is important to note that the model equations refer to the non‐inertial system, therefore, the linear and angular acceleration must be resolved into this system. This is done by using

$$\ddot{\vec{\Omega }}(t)=M(t)\;{\ddot{\vec{\Omega }}}_{I}(t),$$

where ${\ddot{\vec{\Omega }}}_{I}(t)$ is the angular acceleration referred to the inertial system (for example the clinician who makes the maneuvers) and *M*(*t*) a rotation matrix. Since these maneuvers consist of a pitch (y‐axis) followed by a roll movement (x‐axis), the vector ${\vec{\Omega }}_{I}$ for the pitch is (0,*Ω*(*t*),0)^*T*^ and for the roll it is (*Ω*(*t*),0,0)^*T*^.

The rotation matrix for the first turn is

$$M_{1}(t)=\left(\begin{array}{ccc} \cos\Omega (t) & \;\;0 & \;\;\sin\Omega (t)\\ 0 & \;\;1 & \;\;0\\ -\sin\Omega (t) & \;\;0 & \;\;\cos\Omega (t) \end{array},\;\right)\;t\leq \mathrm{\Delta t.}$$

and for the second turn,

$$M_{2}(t)=\left(\begin{array}{ccc} 1 & \;\;0 & \;\;0\\ 0 & \;\;\cos\Omega (t) & \;\;-\sin\Omega (t)\\ 0 & \;\;\sin\Omega (t) & \;\;\;\cos\Omega (t) \end{array},\;\right)\;\left(\begin{array}{ccc} \cos\alpha & \;\;0 & \;\;\sin\alpha \\ 0 & \;\;1 & \;\;0\\ -\sin\alpha & \;\;0 & \;\;\cos\alpha \end{array},\;\right)\;\mathrm{\Delta t}<t\leq 2\mathrm{\Delta t.}$$

Once boundary conditions for the angles are imposed, *Ω*(*t*) is written as follows,

$$\Omega (t)=\left\{\begin{array}{ll} \Omega_{1}(t)=\frac{3\alpha }{\Delta t^{2}}t^{2}-\frac{2\alpha }{\Delta t^{3}}t^{3}, & \;\;\;t\leq \mathrm{\Delta t},\\ \Omega_{2}(t)=5\alpha -\frac{12\alpha }{\mathrm{\Delta t}}t+\frac{9\alpha }{\Delta t^{2}}t^{2}-\frac{2\alpha }{\Delta t^{3}}t^{3}, & \;\;\;\mathrm{\Delta t}<t\leq 2\mathrm{\Delta t.} \end{array}\right)$$

In order to obtain the expressions for the forcing terms *F*_*i*_ and *F*_*n*_, we assume that the geometry of the posterior canal is described by a circle of radius *r*. Therefore,

$$\begin{array}{l} F_{i}=\overset{l_{n}}{\underset{0}{\int }}\rho \;(\ddot{\vec{\Omega }}(t)\wedge \vec{R}(s))\;\vec{\text{d}s}=\rho \;r\;{\ddot{\Omega }}_{2}(t)\sin\Omega_{2}(t)\;l_{n}=\\ \;=\rho \;r\;l_{n}\;\sin(5\alpha -\frac{12\alpha }{\mathrm{\Delta t}}t+\frac{9\alpha }{\Delta t^{2}}t^{2}-\frac{2\alpha }{\Delta t^{3}}t^{3})\;\left(\frac{18\alpha }{\Delta t^{2}}-\frac{12\alpha }{\Delta t^{3}}t\right), \end{array}$$

and

$$\begin{array}{l} F_{n}=\;\frac{a^{2}\;N}{b^{2}}\left[\frac{4}{3}a(\rho_{s}-\rho_{e})\left(-r\;\sin\alpha \;\sin\Omega_{2}(t)\;\;{\ddot{\Omega }}_{2}(t)-g\;\cos(\xi /r)\right)+\frac{6\;\mu \;\dot{\xi }}{a}\right]\\ \;+\;\frac{a\;N\;6\;\mu_{e}}{\Pi \;b^{2}\;(b^{2}-a^{2})}\;\dot{Q}\;, \end{array}$$

where *A*_*s*_=*Πa*^2^, *A*_*e*_=*Πb*^2^, *b* stands for the radius of the cross‐sectional area of the canal and $\dot{\xi }=0.02\;\text{cm}/s$[2].

After imposing the initial condition, the solution of equations (6) is written as

$$Q(t,\alpha ,\theta )=\frac{\exp\{-\theta_{2}\;t/c(\theta_{1},t)\}}{c(\theta_{1},t)}\overset{t}{\underset{0}{\int }}\exp\{\theta_{2}\;s/c(\theta_{1},s)\}F(s,\alpha )\text{d}s,$$

where *F*(*t*,*α*)=*F*_*i*_+*F*_*n*_ and $\;c(\theta_{1},t)=\frac{a\;N\;6\;\mu_{e}}{\Pi \;b^{2}\;(b^{2}-a^{2})}\;\dot{Q}+\theta_{1}$.

## Optimal experimental designs

**Figure 2:**
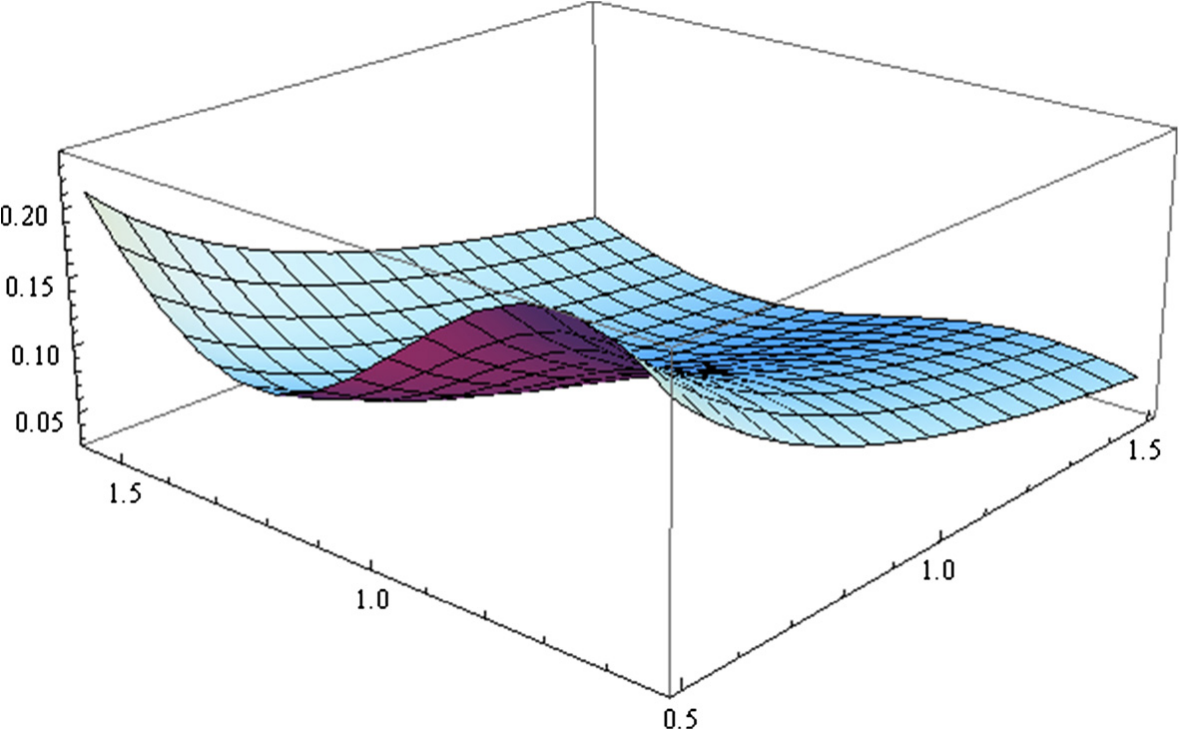
**Plot of the function model.** Plot of the function given by the response *Q*(*Δt*,*α*,*θ*). This is plotted as a function of *Δt* and *α* for the given nominal values $\theta_{1}^{\left(0\right)}=0.85$ and $\theta_{2}^{\left(0\right)}=0.2$.

Optimal designs for the model given in (5) with nominal values of the parameters $\theta_{1}^{\left(0\right)}=0.85$ and $\theta_{2}^{\left(0\right)}=0.2$ (obtained from [15]) are calculated. It is assumed that six particles are detached from the canal wall [16]. On the other hand, it is known that *A*_*s*_/*A*_*e*_ ≈ 10^−4^ and that, approximately, the values of *r* and *l*_*n*_ are 0.1 cm and 0.5 cm, respectively [17]. The values used for the constants are listed in Table 1. Figure 2 shows the plot of the function given by the above mentioned *Q*(*Δt*,*α*,*θ*). This is plotted as a function of *Δt* and *α* for the given nominal values.

**Table 1:** Physical parameters

**xx‐xx Parameter**	**xx‐xx Value**
*A*_*s*_ : Frontal area of the particle	3.14×10^−4^*cm*^2^
*ρ*: density of the canal	1.0 *gcm*^−3^
*ρ*_*s*_: density of the particle	2.7 *gcm*^−3^
*ρ*_*e*_: density of the endolymph	1.0 *gcm*^−3^
*μ*_*e*_: viscosity of the endolymph	8.5×10^−3^*dyns*^−1^*cm*^−1^
*g*: gravitational acceleration	981 *cms*^−2^

The Information Matrix is expressed as

$$M(\xi ,\theta )=\underset{\mathrm{\Delta t},\alpha \in \chi }{\sum }\xi (z)\;f(\mathrm{\Delta t},\alpha ,\theta )f^{T}(\mathrm{\Delta t},\alpha ,\theta ),$$

where

$$f^{T}\;(\mathrm{\Delta t},\alpha ,\theta )=\left(f_{1}(\mathrm{\Delta t},\alpha ,\theta ),f_{2}(\mathrm{\Delta t},\alpha ,\theta )\right)=\left(\frac{\mathrm{\partial Q}(\mathrm{\Delta t},\alpha ,\theta )}{\partial \theta_{1}},\frac{\mathrm{\partial Q}(\mathrm{\Delta t},\alpha ,\theta )}{\partial \theta_{2}}\right).$$

The two components are written as

$$\begin{array}{ll} f_{1}(\mathrm{\Delta t},\alpha ,\theta )=\left(\frac{\partial }{\mathrm{\partial t}}\frac{\exp\{-\theta_{2}\;\mathrm{\Delta t}/c(\theta_{1},\mathrm{\Delta t})\}}{c(\theta_{1},\mathrm{\Delta t})}\right)\cdot \overset{\mathrm{\Delta t}}{\underset{0}{\int }}\exp\{\theta_{2}\;s/c(\theta_{1},s)\}F(s,\alpha )\text{d}s\;+ & \;\\ \;\frac{1}{c(\theta_{1},\mathrm{\Delta t})}\;\frac{\exp\{-\theta_{2}\;\mathrm{\Delta t}/c(\theta_{1},\mathrm{\Delta t})\}}{c(\theta_{1},\mathrm{\Delta t})}\;\overset{\mathrm{\Delta t}}{\underset{0}{\int }}-\frac{\theta_{2}\;s}{c^{2}(\theta_{1},s)}\;\exp\{\theta_{2}\;s/c(\theta_{1},s)\}F(s,\alpha )\text{d}\mathrm{s.} & \; \end{array}$$

and

$$\begin{array}{ll} f_{2}(\mathrm{\Delta t},\alpha ,\theta )=-\frac{\mathrm{\Delta t}\exp\{-\theta_{2}\;\mathrm{\Delta t}/c(\theta_{1},\mathrm{\Delta t})\}}{c^{2}(\theta_{1},\mathrm{\Delta t})}\;\overset{\mathrm{\Delta t}}{\underset{0}{\int }}\exp\{\theta_{2}\;s/c(\theta_{1},s)\}F(s,\alpha )\text{d}s\;+ & \;\\ \;\frac{1}{c(\theta_{1},\mathrm{\Delta t})}\;\exp\{-\theta_{2}\;\mathrm{\Delta t}/c(\theta_{1},\mathrm{\Delta t})\}\;\overset{\mathrm{\Delta t}}{\underset{0}{\int }}\frac{s}{c(\theta_{1},s)}\;\exp\{\theta_{2}\;s/c(\theta_{1},s)\}F(s,\alpha )\text{d}\mathrm{s.} & \; \end{array}$$

### D‐optimal design for a discrete design space

D‐optimal designs for model (5) are obtained in order to estimate simultaneously the parameters, *θ*_1_ and *θ*_2_. Points (*α*,*Δt*) are taken from the design space [0.5,1.5]×[*Π*/6,*Π*/2]. Since it is more realistic to ask the clinician to carry out the maneuvers for typical values of angles and times, a finite design space is being considered, that is,

$$\begin{array}{l} \mathrm{\Delta t}\in \{0.5,\;1,\;1.5\}\;\;\text{and}\\ \alpha \in \{\Pi /6,\;\Pi /4,\;\Pi /3,\;5\Pi /12,\;\Pi /2\}\;=\;\{30\text{°},\;45\text{°},\;60\text{°},\;75\text{°},\;90\text{°}\}. \end{array}$$

Taking this finite set into consideration and maximizing the determinant of the Information Matrix, the optimal design is

$$\xi_{1}^{*}=\left\{\begin{array}{lll} \left(\;1.5,\;\Pi /6\;\right) & \left(\;0.5,\;\Pi /4\;\right) & \left(\;0.5,\;\Pi /2\;\right)\\ \;0.47 & \;0.16 & \;0.37 \end{array},\;\right\}.$$

Table 2 shows that all the values of the generalized variance are smaller than the number of parameters, and therefore the design obtained satisfies numerically the Equivalence Theorem. The design indicates the duration and angle of the maneuvers to be carried out, but not how many observations the sample will have. That will be determined by the researcher through other means (for instance, the budget). The weights will give us the proportion of different maneuvers to be performed. For instance, if the sample contains *N*=100 observations, the clinician will have to repeat the maneuver consisting of a positive 90° pitch from the upright position followed by a positive 90° roll, both with a duration of half a second, 37 times.

**Table 2:** Values of the generalized variance

	*Π*/6	*Π*/4	*Π*/3	5*Π*/12	*Π*/2
0.5	1.96	**2**	1.24	1.53	**2**
1	1.93	1.34	0.19	0.45	0.4
1.5	**2**	1.38	0.06	0.29	0.24

#### Sensitivity analysis of the D‐optimal design

The goodness of any design *ξ* is measured by its efficiency. We will see how efficient the D‐optimal design $\xi_{1}^{*}$ is by checking the value of

(7) $${\text{eff}}_{\theta }(\xi_{1}^{*})=\frac{\Phi_{D}[M(\xi_{\theta }^{*},\theta )]}{\Phi_{D}[M(\xi_{1}^{*},\theta )]}=\frac{{\left[\det M(\overset{*}{\underset{\theta }{\xi }},\theta )\right]}^{-1/m}}{{\left[\det M(\overset{*}{\underset{1}{\xi }},\theta^{\left(0\right)})\right]}^{-1/m}},$$

where $\xi_{\theta }^{*}$ is the D‐optimal design calculated for a possible real value of the parameter *θ*. This efficiency shows how robust the design is with respect to the true (unknown) value of the parameters. To check the robustness of a design, we want to check how the quality of the estimation would be affected by a wrong choice of the nominal value. The efficiency can sometimes be multiplied by 100 and be reported in percentage terms. If, for instance, we take as nominal value *θ*^(0)^=0.5, the true value being *θ*=0.6 and the efficiency being 50%, then the design $\xi_{\theta^{\left(0\right)}}^{*}$ would need to double the total number of observations to perform as well as the optimal design calculated with the true value *θ*=0.6. Thus, our design would not be very robust. Table 3 illustrates the sensitivity of the D‐optimal design with respect to the choice of the parameters *θ*_1_ (horizontal values) and *θ*_2_ (vertical values), i.e, how robust the design is with respect to the true values of those parameters. As can be observed, small variations of the nominal values does not affect the quality of the estimation much.

**Table 3:** Values of the efficiency

	0.1	0.7	**xx‐xx 0.85**	0.9	2
0.015	37 %	98%	98%	78%	28%
**0.2**	35%	97%	**100%**	80%	31%
0.9	29%	84%	90%	80%	30

The generalized variance for the design $\xi_{2}^{*}$ verifies the Equivalence Theorem.

### D‐optimal design for a continuous design space

If instead of the finite set

χ={Π/6,Π/4,Π/3,5Π/12,Π/2}×{0.5,1,1.5},

we would have chosen a continuous design space *χ*=[0.5,1.5]×[*Π*/6,*Π*/2], the D‐optimal design obtained would have been

$$\xi_{2}^{*}=\left\{\begin{array}{lll} \left(\;1.5,\;0.55\;\right) & \;(0.5,\;0.72\;) & \;(0.5,\;\Pi /2\;)\\ \;0.47 & \;0.16 & \;0.37 \end{array},\;\right\}.$$

**Figure 3:**
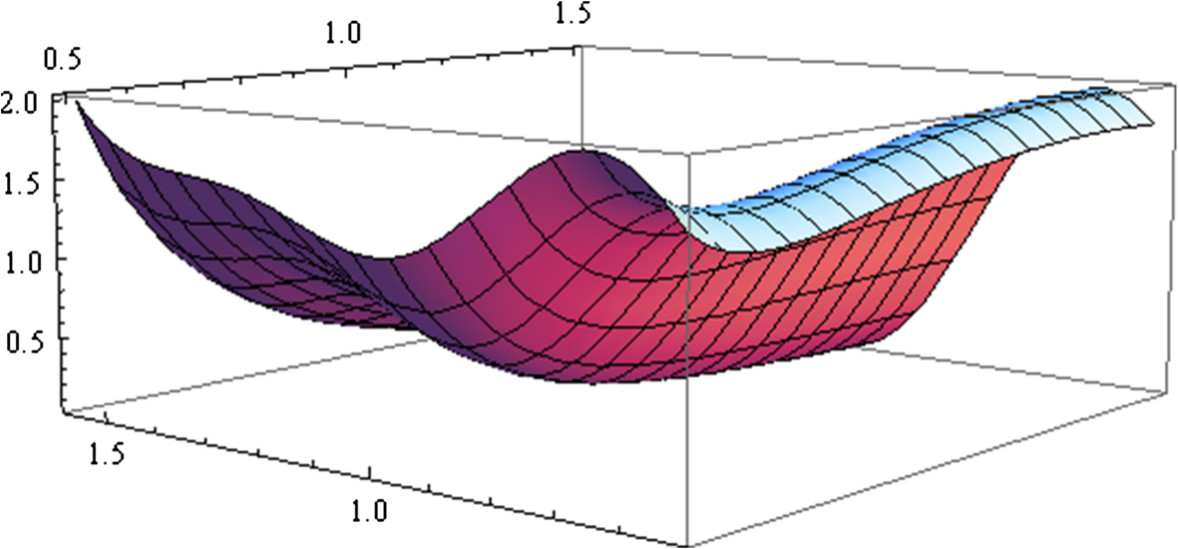
**Plot of the generalized variance.** Plot of the generalized variance for the design obtained. This design will be D‐optimal to estimate both parameters *θ*_1_ and *θ*_2_, if and only if, it verifies the Equivalence Theorem. That is, for all the values of *Δt* and *α* within the space design, the values of the generalized variance must be lower than or equal to than the number of parameters to estimate. In the points of the design, the value of the function must be equal to the number of parameters.

As can be observed, the results would have been very similar to *ξ*_1_, having an efficiency of around 95%. But in practice, the clinician cannot carry out any turns that are smaller than 15° or shorter than half a second. Figure 3 shows how the generalized variance function for a continuous design space satisfies the Equivalence Theorem, that is, for all the values of *Δt* and *α* within the space design, the values of the generalized variance must be lower or equal to the number of parameters to estimate. At the points of the design, the value of the function must be equal to the number of parameters.

### c‐optimal design for a discrete design space

If we are interested in estimating a linear combination of the parameters, say *c*^*T*^*θ*, then we use the *c*‐optimality criterion. An elegant way for finding an optimal design that estimates a linear combination of the parameters was given by Elfving [18]. This method is nicely illustrated and explained, e.g. by Chernoff [19], Kitsos [20] or Wiley[10].

For a given regression problem with regression function *f*(*Δt*,*α*,*θ*), the method first defines the Elfving’s set given by set G, the convex hull of {*f*(*Δt*,*α*,*θ*^(0)^)∪−*f*(*Δt*,*α*,*θ*^(0)^)}, *θ*^(0)^ being a nominal value for the parameter *θ*. This means that the set G is the smallest convex set containing {*f*(*Δt*,*α*,*θ*^(0)^)∪−*f*(*Δt*,*α*,*θ*^(0)^)}. The point of intersection of the straight line defined by the vector *c* with the boundary of the Elfving’s set determines the *c*‐optimal design, *ξ*^∗^, as a convex combination of the vertices of G. These vertices provide the support points of the optimal design. The weights in the convex combinations are the weights of the optimal design. Furthermore, *Φ*_*c*_(*ξ*^∗^)=*c*^*T*^*M*^−1^(*ξ*,*θ*) *c*=(∥ *c* ∥ / ∥ *c*^∗^ ∥)^2^, where *c*^∗^ is the vector defined by the cut point of the straight line defined by *c* with the boundary of G.

**Figure 4:**
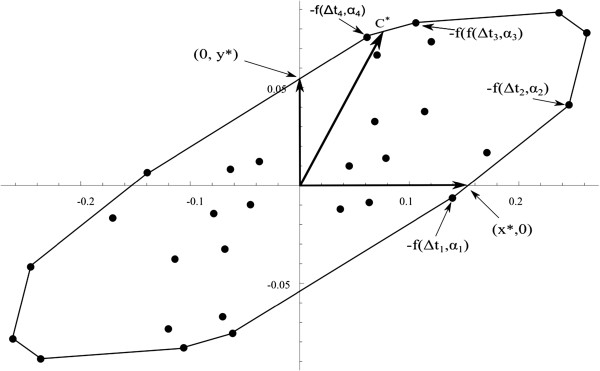
**Elfving’s set for a discrete design space.** Plot of the Elfving’s set for a discrete design space. This is used to find the *c*‐optimal design to estimate a linear combination of the parameters.

Considering the same finite set as before, the interest is again in estimating one parameter, but now both of them are unknown. Elfving’s set is displayed in Figure 4. In order to estimate the parameter *θ*_1_, let *c*^*T*^=(1,0). The straight line defined by this vector cuts the boundary of the Elfving’s set at point (*x*^∗^,0) and it defines the $c_{\theta_{1}}$‐optimal design to estimate the parameter *θ*_1_. This point is expressed as a convex combination of the points −*f*(*Δt*_1_,*α*_1_)=−*f*(0.5,5*Π*/12) and −*f*(*Δt*_2_,*α*_2_)=−*f*(0.5,*Π*/2) with weights *p*_1_=0.86 and *p*_2_=0.14, respectively. Therefore, the optimal design is

$$\xi_{3}^{*}=\left\{\begin{array}{ll} \left(\;0.5,\;5\Pi /12\;\right) & \;\;(\;0.5,\;\Pi /2\;)\\ 0.86 & \;0.14 \end{array},\;\right\}.$$

On the other hand, to estimate the parameter *θ*_2_, let *c*^*T*^=(0,1). The straight line defined by this vector cuts the boundary of the Elfving’s set at point (0,*y*^∗^), and it defines the $c_{\theta_{2}}$‐optimal design to estimate the parameter *θ*_2_. This point is expressed as a convex combination of points *f*(*Δt*_1_,*α*_1_) and −*f*(*Δt*_4_,*α*_4_)=−*f*(1.5,*Π*/6) with weights *p*_1_=0.3 and *p*_2_=0.7, respectively. Therefore, the optimal design is

$$\xi_{4}^{*}=\left\{\begin{array}{ll} \left(\;0.5,\;5\Pi /12\;\right) & \;\;(\;1.5,\;\Pi /6\;)\\ 0.3 & \;\;0.7 \end{array},\;\right\}.$$

Finally, to obtain the c‐optimal design for the estimation of *θ*_1_+*θ*_2_ we use *c*^*T*^=(1,1) to define the point *c*^∗^. This point is expressed as a convex combination of the points −*f*(*Δt*_4_,*α*_4_)=−*f*(1.5,5*Π*/12) and −*f*(*Δt*_3_,*α*_3_)=−*f*(1,*Π*/6) with weights *p*_1_=0.8 and *p*_2_=0.2, respectively. So the c‐optimal design is now

$$\xi_{5}^{*}=\left\{\begin{array}{ll} \left(\;1.5,\;5\Pi /12\;\right) & \;\;(\;1,\;\Pi /6\;)\\ 0.8 & \;\;0.2 \end{array},\;\right\}.$$

One important aspect is to check the efficiency of the D‐optimal design $\left(\xi_{D}^{*}\right)$ with respect to the c‐optimal design $\left(\xi_{c}^{*}\right)$. The formula of the efficiency,

(8) $${\text{eff}}_{c}(\xi_{D}^{*})=\frac{c^{T}M^{-1}(\xi_{c}^{*},\theta^{\left(0\right)})c}{c^{T}M^{-1}(\xi_{D}^{*},\theta^{\left(0\right)})c},$$

provides a way to see how good the D‐optimal design is at estimating each of the parameters. In the case of *θ*_1_, the D‐optimal design $\xi_{1}^{*}$ and c‐optimal design $\xi_{3}^{*}$ are compared and the efficiency is around 75%, while for *θ*_2_, $\xi_{1}^{*}$ and $\xi_{4}^{*}$ are compared, having an efficiency of around 85%. These results show the D‐optimal design is more efficient for estimating *θ*_2_ than for estimating *θ*_1_, that is, with this design, the test power for testing {*H*_0_:*θ*_2_=0} will be greater that the test power for {*H*_0_:*θ*_1_=0}.

### c‐optimal design for a continuous design space

**Figure 5:**
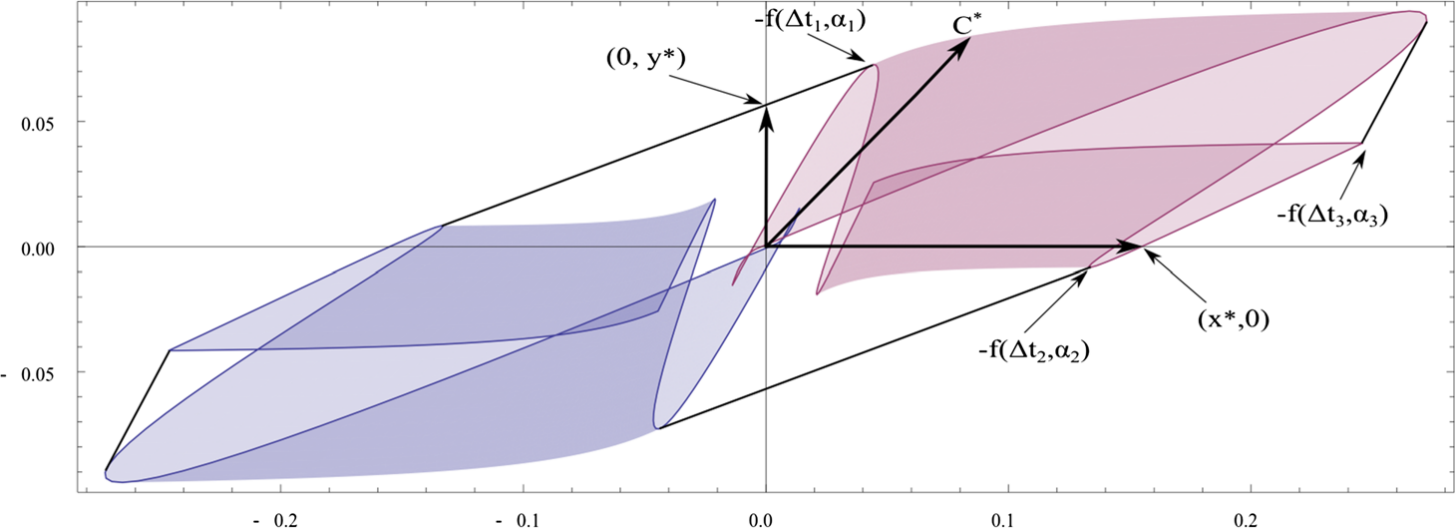
**Elfving’s set for a continuous design space.** Plot of the Elfving’s set for a continuous design space.

For a continuous design space, the Elfving’s set obtained is shown in Figure 5 by plotting the convex hull of the surface {*f*(*Δt*,*α*,*θ*^(0)^)∪−*f*(*Δt*,*α*,*θ*^(0)^)}. In this case *t*∈[0.5,1.5]×[*Π*/6,*Π*/2]. With the help of the method proposed by López‐Fidalgo and Rodríguez‐Díaz [21], we will calculate the c‐optimal design to estimate the parameters *θ*_1_, *θ*_2_ and an example of a linear combination of them, *θ*_1_+*θ*_2_.

The straight line defined by the vector *c*^*T*^=(1,0) cuts the Elfving’s set at point (*x*^∗^,0)=(0.144, 0) and it defines the $c_{\theta_{1}}$‐optimal design to estimate the parameter *θ*_1_. This point is expressed as a convex combination of −*f*(*Δt*_2_,*α*_2_)=−*f*(0.49,1.29) and −*f*(*Δt*_3_,*α*_3_)=−*f*(0.5,1.57) with weights *p*_1_=0.8 and *p*_2_=0.2, respectively. Therefore, the optimal design is

$$\xi_{6}^{*}=\left\{\begin{array}{ll} \left(\;0.49,\;73.9\text{°}\;\right) & \;\;(\;0.5,\;89\text{°}\;)\\ 0.8 & \;\;0.2 \end{array},\;\right\}.$$

The straight line defined by vector *c*^*T*^=(0,1) cuts the boundary of the Elfving’s set at point (0,*y*^∗^)=(0, 0.057) and it defines the $c_{\theta_{2}}$‐optimal design to estimate the parameter *θ*_2_. This point is expressed as a convex combination of −*f*(*Δt*_1_,*α*_1_)=−*f*(1.5,0.49) and −*f*(*Δt*_2_,*α*_2_)=−*f*(0.49,1.29) with weights *p*_1_=0.75 and *p*_2_=0.25, respectively. Therefore, the optimal design is

$$\xi_{7}^{*}=\left\{\begin{array}{ll} \left(\;0.49,\;73.9\text{°}\;\right) & \;\;(\;1.5,\;28\text{°}\;)\\ 0.75 & \;\;0.25 \end{array},\;\right\}.$$

The efficiency of the D‐optimal design for estimating the parameter *θ*_1_ is around 75%, while for *θ*_2_, it is around 90%. Finally, to obtain the c‐optimal design for the estimation of *θ*_1_+*θ*_2_, vector *c*^*T*^=(1,1) is considered. In this case, the straight line defines the point *c*^∗^=(0.084,0.084)=−*f*(1.3,0.6), so the c‐optimal design is supported at one point:

$$\xi_{8}^{*}=\left\{\begin{array}{l} \left(1.3,\;34\text{°}\right)\\ 1 \end{array}\right\}.$$

As we can observe, the results obtained are quite similar to the case concerning the discrete design space, except for the estimation of *θ*_1_+*θ*_2_, where for the continuous case the *c*‐optimal design is only supported at one point, although in both cases the angle of turn is similar. If the number of support points in a c‐optimal design is less than the number of parameters, this design allows the computation of the maximum likelihood estimate of this linear combination. But, in this case, not all the parameters are identifiable individually, that is, some of them cannot be estimated.

## Discussion

The present biomechanical model was derived to provide a quantitative basis for the detection of BPPV. This model is based on a maneuver consisting of two consecutive head turns. These are the most common head movements leading to vertigo symptoms. We would like to remark that although the model can only be applied for this specific maneuver, it could be extended to other types. The experiment, that is, the duration and angle of the head movements to be applied to the patients should be based on the design provided by the D‐optimal design since it helps us estimate the parameters simultaneously, minimizing the confidence ellipsoid. The c‐optimal design is used either for estimating linear combinations of the parameters, or for estimating the parameters separately. But in this case, it also provides valuable assistance to check how efficient the D‐optimal design is for the estimation of each of the parameters. This is an interesting check of the sensitivity since a D‐optimal design could be quite efficient for estimating a particular parameter but quite inefficient for estimating another one.

The covariance matrix of the estimates is asymptotically proportional to the inverse of the Fisher Information Matrix. Theoretically, the application of the maneuvers which are specified in the design, along with their corresponding proportions, will assure that an objective function of the covariance matrix of the estimators

$$\Sigma_{\hat{\theta }}=\left(\begin{array}{ll} \text{var}\;\;{\hat{\theta }}_{1} & \text{cov}\;({\hat{\theta }}_{1},{\hat{\theta }}_{2})\\ \text{cov}\;({\hat{\theta }}_{1},{\hat{\theta }}_{2}) & \text{var}\;\;{\hat{\theta }}_{2} \end{array}\right)\propto M^{-1}(\xi ,\theta ),$$

will be minimized. Symbol ∝ stands for “asymptotically proportional” in this case.

Finally, we would like to point out that, as far as the authors know, the models found in the published works describing this sort of maneuvers have not been validated with data yet. The clinicians hold that the extra volume of endolymph displaced by the otoliths are directly related to the eye movements provoked in the patient under vertigo symptoms. Therefore, in some way, to validate the model, response *y* should be measured through some variable related to eye movement.

## Competing interests

The authors declare that there are no potential competing interests in our paper.

## Authors’ contributions

SC carried out the derivation of the mathematical model, performed the computations of the designs and drafted the manuscript. JL provided the main theoretical ideas in optimal design, supervised the whole process and helped to draft and correct the manuscript. All authors read and approved the final manuscript.

## Authors’ information

S. Campos is pursuing a PhD under the supervision of Prof. López‐Fidalgo who is Professor of Statistics and Dean of the Industrial Engineering School of the University of Castilla‐La Mancha (Spain). He is an ISI Elected Member and holds Visiting Positions at the University of Manchester, Institute of Sciences and Technology (UMIST), the University of California, Los Angeles (UCLA) and the University of California, Riverside (UCR). Prof. López‐Fidalgo has written numerous academic publications; he is the former Editor of the Bulletin of the Spanish Statistical Society, Editor of the proceedings MODA8 (Springer), Associate Editor of Test and Sankhya B. He has published more than 70 papers, in such publications as the Journal of the American Statistical Association, Journal of the Royal Statistical Society, series B, Bioinformatics or Pharmaceutical Statistics, amongst others. Prof. López‐Fidalgo has also been representative of the Spanish Agency of Research for Mathematics.
